# Obesity: a gender-view

**DOI:** 10.1007/s40618-023-02196-z

**Published:** 2023-09-23

**Authors:** G. Muscogiuri, L. Verde, C. Vetrani, L. Barrea, S. Savastano, A. Colao

**Affiliations:** 1 Dipartimento Di Medicina Clinica E Chirurgia, Diabetologia E Andrologia, Unità Di Endocrinologia, Università Degli Studi Di Napoli Federico II, Via Sergio Pansini 5, 80131 Naples, Italy; 2 Centro Italiano Per La Cura E Il Benessere del Paziente Con Obesità (C.I.B.O), Dipartimento Di Medicina Clinica E Chirurgia, Diabetologia E Andrologia, Unità Di Endocrinologia, Università Degli Studi Di Napoli Federico II, Via Sergio Pansini 5, 80131 Naples, Italy; 3 Cattedra Unesco “Educazione Alla Salute E Allo Sviluppo Sostenibile”, University Federico II, Naples, Italy; 4 Department of Public Health, University of Naples Federico II, Naples, Italy; 5 Dipartimento Di Scienze Umanistiche, Centro Direzionale, Università Telematica Pegaso, Via PorzioIsola F2, 80143 Naples, Italy

**Keywords:** Gender, Obesity, Body composition, Diet, Weight loss drugs

## Abstract

**Purpose:**

There is a growing awareness of the importance of understanding gender differences in obesity. The aim of this short review was to revise the current evidence on anthropometric characteristics and nutritional and pharmacological aspects of obesity from a gender perspective.

**Methods:**

A literature search within PubMed was performed. Selected publications related to obesity and gender differences were reviewed.

**Results:**

The prevalence of obesity among men is higher than in women, but women have a higher percentage of body fat content compared to men, and gender appears to be an important factor in the manifestation of central (android) or peripheral (gynoid) obesity. In addition, while in most clinical trials, women are still underrepresented, in clinical registration trials of anti-obesity drugs, women are commonly up-represented and gender-specific analysis is uncommon. Considering that adipose tissue is one of the factors affecting the volume of distribution of many drugs, mainly lipophilic drugs, gender differences might be expected in the pharmacokinetics and pharmacodynamics of anti-obesity drugs. Indeed, although Liraglutide 3 mg, a long-acting glucagon-like peptide-1 receptor agonist, and naltrexone/bupropion display lipophilic properties, currently, a gender–dose adjustment for both these drugs administration is not recommended. In addition, despite that predicted responders to treatment offer substantial opportunities for efficient use, especially of expensive new therapies, such as anti-obesity drugs, data on gender differences to identify early responders to both these have not yet been investigated. Finally, bariatric surgery gender disparity reflects healthcare practices. Weight loss similar, but differing effects: women need more correction and face psychology challenges; men have worse physiology and fewer comorbidity improvements.

**Conclusion:**

Gender differences exist in obesity prevalence and phenotype, body fat distribution, drug efficacy, clinical trial representation, and different secondary effects of bariatric surgery. Gender is an important variable in obesity analysis.

## Introduction

Obesity is one of the major health concerns worldwide, with a multitude of health consequences. According to the National Health and Nutrition Examination Survey (NHANES) 2005–2014, the prevalence of obesity among women is higher than among man, at 40% versus 35%, respectively [1]. According to the Italian Observatory on Healthcare Report (2016), in Italy, up to 45% of adults suffered from overweight or obesity, with a north–south geographical gradient of obesity [2]. Interestingly, in Italy, the prevalence of obesity among men is higher than among women, 51% versus 34%, respectively [2]. This uneven distribution of obesity prevalence between genders in different countries suggests that, in addition to biological and behavioral factors, socio-economic factors may also play a role in determining this phenomenon. Studies have shown that in countries with lower gross domestic product per capita, greater income inequality, and greater sex inequality, the prevalence of obesity tends to be higher among women than among men [3]. This suggests that socio-economic factors also play a significant role in determining gender differences in obesity prevalence.

The difference in prevalence and rate of increase of obesity between men and women led several authors to investigate the gender-related pathogenetic mechanisms and phenotype of obesity and to identify a gender-specific weight-loss approach.

Besides a difference in terms of prevalence, a gender-related adipose tissue distribution exists [4]. For a given body mass index (BMI), there are well-known body composition differences between sexes, including higher fat-free mass in men and higher adiposity in women. Men usually have a central adipose tissue distribution (android), whereas women show a peripheral adipose tissue distribution (gynoid), particularly in the limbs and hips. The higher visceral adiposity observed in men is associated with negative metabolic consequences, such as elevated postprandial insulin, free fatty acids, and triglyceride levels. Conversely, the peripheral adipose tissue distribution, typically found in women, is less associated with the development of obesity-related complications compared to the central adipose tissue distribution [4].

Gender differences in body composition may be due, at least in part, to the effects of sex hormones. It seems that female sex has a favorable effect on insulin sensitivity, despite women having higher adiposity relative to men [5]. This has been suggested by a decreased insulin sensitivity after menopause and subsequent improvement with estrogen therapy, leading to the hypothesis that estrogens might play a role in the insulin sensitivity observed in women [5]. Androgens have gender-specific effect on adipose tissue and insulin resistance [4]. In fact, increased androgen levels have been associated with insulin resistance in women, while hypogonadal states (low testosterone concentration) in men were associated with insulin resistance, and testosterone replacement resulted in an improvement of insulin resistance [4].

Obesity is usually associated with a status of 'low-grade inflammation', and it has been shown in mice that males and females differ in the inflammatory response that occurs in obesity [6]. Indeed, it has been observed that males show increased infiltration of M1 (CD11c +) macrophages in adipose tissue, while females exhibit increased infiltration of M2 (CD11c-) macrophages in models of inflammatory response to a high-fat diet in mice. Additionally, males have higher levels of inflammatory cytokines than females [6]. Research involving bone marrow transplants has suggested that this sex-dependent inflammatory response is linked to inherent differences in the composition or activation of hematopoietic stem cell populations [7]. Indeed, in humans, peripheral blood mononuclear cells from men produce more pro-inflammatory TNF-α and less-protective IL-10 than peripheral blood mononuclear cells from women, following lipopolysaccharide stimulation [7]. Autoimmune diseases are among the most strongly sex-biased of all diseases, and 80% of autoimmune patients are women [8]. In this regard, estrogens and androgens exert distinct influences over cytokine production and T-cell differentiation [9, 10]. Testosterone is suppressive of both the adaptive and innate immune responses [10], whereas estrogen may promote inflammatory responses [9].

Furthermore, overwhelming evidence suggests a gender-specific food intake regulation [11]. In fact, plasma concentrations of the adipocyte-derived anorexigenic hormone leptin, which plays an important role in the regulation of food intake and body weight, have been reported to be up to four times higher in women than in men [12]. This difference is likely due to the fact that women typically have a higher percentage of body fat. Additionally, leptin levels are linked to subcutaneous adipose tissue (SAT), which is known to be associated with a favorable metabolic profile [12]. Adiponectin is a hormone secreted exclusively by adipose tissue, and the reduction in states of insulin resistance appears significantly higher in women than in men, even after adjusting for differences in body composition [13].

In addition to the hormonal patterns, a gender difference in terms of metabolic response to food intake has been demonstrated [14]. Men have significantly higher postprandial insulin, free fatty acids, and triglyceride levels after a standardized meal compared to women [14].

Moreover, evidence has demonstrated a gender relationship between weight-related attitudes and behaviors [15]. Compared with women, men with obesity were less likely to have accurate weight perception, weight dissatisfaction, or attempted weight loss. Further, it has been shown how women and men usually choose different weight-loss strategies: men attempt weight loss, are more likely to exercise and eat less fat, whereas women are more to likely join a weight-loss program, take diet pills or prescription, follow a special diet, and eat more vegetables and fruits [15].

Gender-related differences in the response to obesity treatment have been very scarcely investigated in the past, and, consequently, current therapeutic interventions for obesity are not gender-adjusted. A few studies explored gender-related differences in the response to orlistat [16, 17]. It has been reported that although this drug causes similar weight loss in men and women, the psychological response to the unpleasant gastrointestinal side effects of the therapy is worse in women, which could reduce adherence to therapy [16, 17]. Tchoukhine et al. (2011) reported that orlistat was effective only in men and not in women to reduce the excess body weight induced by treatment with clozapine and olanzapine [18]. Conversely, data from the Review Of MEn and Obesity (ROMEO) project suggested that orlistat could be less beneficial in men than in women for weight maintenance [16]. In the last few years, interest in drug therapy for obesity flared up again when a heterogeneous group of new anti-obesity drugs became available for use in humans. Only two of them have been approved by the EMA: liraglutide 3 mg and naltrexone/bupropion, both in 2015 [19]. However, no evidence is currently available to promote a gender–adjustment dose of administration. Thus, the aim of this manuscript was to revise the current evidence on anthropometric characteristics, nutritional, pharmacological, and surgical aspects of obesity from a gender perspective.

## Body composition and eating behavior

Men and women present specific differences in body composition, adipose tissue distribution, and metabolism, which begin in intrauterine life, continue throughout childhood, and become apparent during adolescence [20]. The gender difference in adipose tissue deposition relies on a complex interaction between several factors, i.e., genetic determinants, epigenetic mechanisms, specific adipose cell elements, hormonal determinants, and social and environmental influences [21, 22]. Indeed, the combination of sex chromosomes, genetic variability, and environment play a relevant role. At fertilization, the combination of sex chromosomes determines biological sex, with XX for females and XY for males [23]. After the differentiation of gonads, it becomes challenging to distinguish the effects attributed to sex chromosomes from those resulting from sex steroid hormones. The Four Core Genotypes (FCG) mouse model, which involves mice with XX or XY chromosomes on both male and female gonadal backgrounds, is commonly used to study the effects of gonadal and chromosome sex. Notably, XX mice, regardless of their gonadal type (ovaries or testes), exhibit a higher proportion of fat mass, highlighting the significant role of X chromosomes in regulating adiposity [23]. Apart from genetic variation, epigenetic mechanisms may also play a role in the differences between sexes in terms of adiposity and fat distribution [24, 25]. Exposure to harmful factors, such as malnutrition, pollutants, stress, and endocrine disruptors during pregnancy, can lead to the alteration of gene expression, impacting adiposity in adults [24]. Importantly, these effects can differ between women and men [24]. For instance, female offspring of mothers who experienced undernutrition during pregnancy, as seen during the Dutch famine of 1944–1945, have an increased tendency to gain adiposity in adulthood [25]. Conversely, maternal obesity is more likely to result in increased adiposity in male offspring [26].

**Fig. 1:**
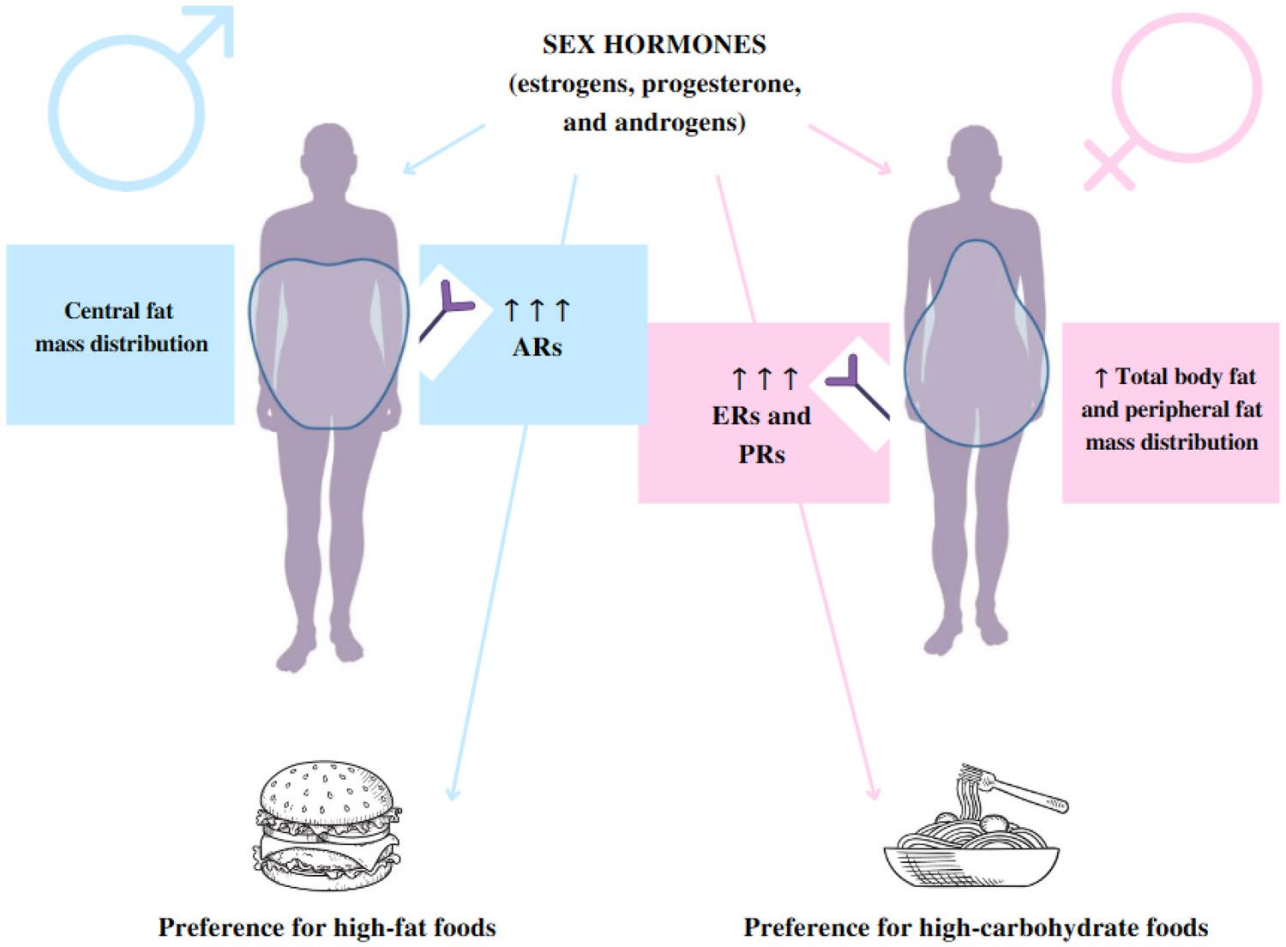
Gender differences in body composition and eating behavior. Subcutaneous adipose tissue presents higher concentrations of estrogen and progesterone receptors than androgen receptors. Conversely, visceral adipose tissue has a higher concentration of androgen receptors. Hormonal differences may explain the sexual dimorphism of adiposity between men and women: women have more adipose tissue than men and a peripheral distribution of fat mass, while men have a central distribution of fat mass. Sex hormones also influence food preferences: women prefer more carbohydrate-rich and sweet foods, while men prefer more fat-rich foods. *ARs* androgen receptors; *ERs* estrogen receptors;* PRs* progesterone receptors

Among other factors, sex hormones (estrogens, progesterone, and androgens) play a dominant role in these differentiation processes, particularly during adolescence [20]. Indeed, sex hormone receptors are expressed in both visceral adipose tissue (VAT) and SAT [27]. SAT presents higher concentrations of estrogens and progesterone receptors than androgen receptors. Conversely, VAT has a higher concentration of androgen receptors [27]. Therefore, in women, the increased estrogen levels activate the sympathetic system and downregulate androgen receptor expression in the SAT, favoring fat deposition [28]. In men, the rise of testosterone during puberty increases lipolysis and protein synthesis [29]. These hormonal differences can explain the sexual dimorphism of adiposity, with women having more adipose tissue than men (Fig. 1) [27].

In addition, sex hormones influence adipose tissue distribution and body shape. In women, adipose tissue is mainly present in the lower part of the body (hips and thighs), whereas men have an abdominal and upper thoracic distribution of adipose tissue [27]. Estrogen reduction during menopause is associated with the shift from gluteofemoral to central adipose tissue accumulation in women [30]. Notably, several studies have demonstrated that hormone replacement therapy (HRT) in postmenopausal women reduces fat mass accumulation as compared to postmenopausal women with no treatment [30–32]. Kristensen et al. observed that a 5-year HRT treatment significantly reduced fat mass deposition, especially in the trunk region, in 89 postmenopausal women treated with HRT compared with 178 controls [32]. In line with these results, Ahtiainen et al., in a study considering 10 pairs of monozygotic twins, demonstrated that postmenopausal women on HRT (2–10 years) presented a lower waist circumference ( – 6 cm) than their sisters who did not undergo HRT [33]. More recently, the study by Costa et al. in 32 postmenopausal women confirmed that HRT can significantly reduce visceral adipose tissue deposition [31].

As for body composition, the amount of fat- and fat-free mass differs among men and women [34]. It has been shown that, given the same BMI, women have a 10% higher fat mass compared to men [34]. Recently, Schorr et al. investigated sex differences in body composition using several imaging techniques in 94 men and 114 women with overweight obesity [35]. This study confirmed that women presented a higher total fat mass than men, with fat storage in SAT and VAT, respectively. However, the evaluation of ectopic adipose tissue deposition showed that men are more prone to intramyocellular and intrahepatic lipid storage. Conversely, women had more lower extremity adipose tissue deposition (i.e., lipedema) [35]. In particular, lipedema is a chronic, progressive disease characterized by abnormal fat accumulation leading to painful limbs. It can lead to considerable disability, impairment of daily activities, and psychosocial distress [36]. Therefore, this condition might also influence the eating behavior of women with lipedema.

Interestingly, studies on transgender individuals provide more evidence on the pivotal role of sex hormones as the main determinants of body composition [37]. Indeed, a meta-analysis by Klaver et al., considering 171 transwomen (male-to-female gender) and 354 transmen (female-to-male gender), showed that cross-sex hormone therapy significantly affected body composition. In particular, in transwomen, fat mass increased (+ 3.0 kg), while fat-free mass decreased (– 2.4 kg). Conversely, a reduction in fat mass (– 2.6 kg) and an increase in fat-free mass (+ 3.9 kg) were detected in transmen [37].

Sex hormones have been shown to influence eating behavior, in the so-called “homeostatic” control of energy intake as well as “the hedonic” control of food intake [38, 39]. Physiologically, estrogens influence food intake by both central (i.e., hypothalamic circuits) and peripheral signals. Indeed, many studies in mice have demonstrated that reduced estrogen levels may induce hyperphagia [39]. Conversely, gonadectomized males reduce food intake [38]. In humans, a recent study by Krishnan et al. investigated the association between eating behavior and menstrual cycle in 17 premenopausal women [40]. The luteal phase was associated with a craving for carbohydrate and sweet-rich foods and activation of the endocannabinoid system. No significant association between the follicular phase and eating habits was found [40]. A recent study by Stea et al. investigated the sex-based differences in food choices with a cross-sectional study in 21 European countries (*n* = 37,672 individuals) [41]. The results showed that women (*n* = 19,815) presented a higher consumption of fruit and vegetables than men (*n* = 17,857) [41]. In line with these results, the study by Wardle et al. in 19,298 individuals (8482 men and 10816 women) reported that fruit and fiber intake was higher in women than in men [42]. In addition, men consumed more high-fat foods and salt. Interestingly, the authors also evaluated the main triggers for food choices. Dieting and the need for a healthier diet represented the main drivers behind women’s preferences [42].

Food choices and overeating have been consistently associated with emotional or stress-related eating [43]. In particular, O’Connor et al., in a group of 466 workers (215 men and 251 women), detected a high rate of snack consumption following stressor conditions (interpersonal and work-related issues) [44]. As for the type of snack, a direct correlation between high-fat or sugary foods and stressor conditions was found only in women [44]. Therefore, these data suggest that eating habits are influenced by sex hormones (Fig. 1). However, the role of emotion and beliefs should also be evaluated in the assessment of sex differences in eating behaviors.

## Gender perspective of anti-obesity pharmacological treatment

The FDA approved five drugs for weight loss in obesity: orlistat, phentermine–topiramate, naltrexone-bupropion, liraglutide, and semaglutide [45]. Clinical experience on the ground with these new drugs is still limited, and real-world evidence on their effectiveness and tolerability is eagerly awaited. In particular, it is still unclear how much gender differences impact their clinical effectiveness, although gender-sensitive steps do exist in the pharmacokinetics and in the pharmacodynamics of both of these medicines. Two main mechanisms account for the anorexigenic effect of liraglutide: a central anorexigenic effect and the inhibition of gastric emptying. The central anorexigenic effect is exerted on brain regions involved in feeding behavior, such as the arcuate nucleus and the central reward circuitry [46]. Interestingly, in rodents, the effect of GLP-1 on central reward pathways is critically modulated by estrogens being attenuated by antiestrogens [46]. In preregistration clinical trials, gender-related differences have also been observed in liraglutide disposition, as liraglutide exposure was about 32% higher in women than in men, possibly because of more efficient drug degradation in men [47].

Bupropion strongly affects noradrenergic and dopaminergic neurotransmissions in the brain by blocking the presynaptic transporters for dopamine and noradrenaline [48]. The consequent alterations of noradrenergic and dopaminergic neurotransmission in brain regions involved in feeding control, such as the arcuate nucleus and the mesolimbic system, are responsible for the central anorexigenic effect of bupropion. Naltrexone potently synergizes with bupropion by counteracting an opioid-dependent feedback mechanism that is activated during bupropion treatment and limits the effect of the bupropion drug on POMC neurons in the hypothalamus [48]. Some evidence from preclinical investigations suggests that the central anorexigenic effect of the naltrexone/bupropion combination could be modulated by sex steroids [49]. In particular, it has been shown that estrogens enhance the expression and activity of dopamine transporters both in neurons and in astrocytes [50]. Although the effect of estrogens on the anorexigenic mechanism of bupropion has not been investigated so far, published data demonstrate that these hormones do potentiate its antidepressant activity [51]. In addition, it is known that, upon binding to plasma membrane Gq-mER receptors, estrogens desensitize μ-opioid receptors in anorexigenic hypothalamic POMC neurons [52]. Interestingly, gender-related differences have been reported in humans in the acute response of the hypothalamic–pituitary–adrenal axis to naltrexone and in the clinical response to this drug in alcohol dependence [53]. Gender differences could also exist in the disposition of Mysimba, because sex steroids do affect several steps in the pharmacokinetics of both naltrexone and bupropion. More in detail, bupropion is hydroxylated in the liver by CYP2B6 to hydroxybupropion and CYP2B6 is potently induced by estrogens [54]. Different bupropion and hydroxybupropion pharmacokinetic profiles in males and females have been observed in humans in some studies but not confirmed in others [51, 55, 56]. Naltrexone is converted in the liver into 6α-hydroxynaltrexone by dihydrodiol dehydrogenase (DD1, DD2, and DD4), and testosterone and dihydrotestosterone are the most potent competitive inhibitors of this enzyme [57]. It is currently unknown whether the effect of androgens on dihydrodiol dehydrogenase could cause gender-related differences in naltrexone disposition in humans.

Thus, when considering gender differences in the context of new anti-obesity drugs, it has been hypothesized that physiological and hormonal differences between men and women might impact how these drugs work [49]. However, despite these theoretical possibilities, clinical studies conducted so far have not consistently shown significant differences in the effectiveness or safety of anti-obesity drugs between men and women that would warrant dose adjustments based on gender. This means that, based on the available scientific evidence, the differences between genders in response to these medications have not been substantial enough to require different dosing guidelines for men and women [49].

## Gender perspective of bariatric surgery

Bariatric surgery, a weight-loss procedure, is more commonly undergone by women than men [58, 59]. A study analyzing 61,708 patients over a 10-year period revealed that 22% of bariatric surgeries were performed on men, while 78% were performed on women [59]. Men who undergo bariatric surgery tend to be older, have a higher BMI, and have more comorbidities [59]. Interestingly, despite this gender imbalance, studies have shown that gender is not associated with interest in bariatric surgery or concerns about its cost, benefits, or procedure details among patients seeking weight loss [60, 61]. This suggests that the gender imbalance in bariatric surgery patients may be due to factors such as screening, diagnosis, and counseling practices of healthcare providers, as well as differences in health awareness and perceptions of obesity [62]. As women are more likely to be screened and counseled for weight loss [62], they may also be more likely to be advised to consider bariatric surgery, leading to a higher proportion of surgeries being performed on women.

Regarding outcomes, there are minimal differences in weight loss between men and women after bariatric surgery [58, 63, 64]. However, studies have found significant associations between gender and secondary outcomes. Women are more likely to require corrective procedures following bariatric surgery [65]. Additionally, women tend to experience poorer psychological outcomes, including lower body image, lower psychological well-being, higher rates of depression, and lower satisfaction with the surgery itself [59]. On the other hand, men have worse physiological outcomes and are less likely to see resolution of comorbidities, such as hyperlipidemia, insulin-dependent diabetes, and sleep apnea compared to women [59].

## Conclusion

This literature review highlighted the importance of considering gender as an important variable in the analysis of obesity. The results suggest that men are more likely to develop obesity than women, although there are considerable differences depending on the geographical area and level of economic development of the country. Men and women have specific differences in body composition, adipose tissue distribution, and metabolism, all of which depend to a large extent on different hormonal backgrounds. Sex hormones also seem to influence eating habits between women and men. Of note, gender differences in response to anti-obesity drugs have been found, suggesting that drug therapies may need to be tailored to the sex of the patient. Finally, bariatric surgery, more common in women, may result from healthcare practices and perceptions. Outcomes show similar weight loss but differing secondary effects.
